# Towards a better understanding of the neuro-developmental role of autophagy in sickness and in health

**DOI:** 10.15698/cst2021.07.253

**Published:** 2021-06-29

**Authors:** Juan Zapata-Muñoz, Beatriz Villarejo-Zori, Pablo Largo-Barrientos, Patricia Boya

**Affiliations:** 1 Centro de Investigaciones Biológicas Margarita Salas, CSIC, Madrid, Spain.; 2 Universiteit van Amsterdam, Amsterdam, The Netherlands.

**Keywords:** neurodevelopment, developmental disorders, neuronal autophagy, autism spectrum disorder, neurogenesis

## Abstract

Autophagy is a critical cellular process by which biomolecules and cellular organelles are degraded in an orderly manner inside lysosomes. This process is particularly important in neurons: these post-mitotic cells cannot divide or be easily replaced and are therefore especially sensitive to the accumulation of toxic proteins and damaged organelles. Dysregulation of neuronal autophagy is well documented in a range of neurodegenerative diseases. However, growing evidence indicates that autophagy also critically contributes to neurodevelopmental cellular processes, including neurogenesis, maintenance of neural stem cell homeostasis, differentiation, metabolic reprogramming, and synaptic remodelling. These findings implicate autophagy in neurodevelopmental disorders. In this review we discuss the current understanding of the role of autophagy in neurodevelopment and neurodevelopmental disorders, as well as currently available tools and techniques that can be used to further investigate this association.

## INTRODUCTION

All living organisms and their cells maintain an internal equilibrium between their components, a phenomenon known as ‘homeostasis'. Cellular homeostasis is maintained by tight regulation of the balance between the synthesis and degradation of different cellular components, from macromolecules (e.g. proteins, nucleic acids) to cellular organelles (e.g. ribosomes, mitochondria). Cell homeostasis relies on several catabolic or degradative mechanisms, including autophagy. Derived from the Greek for ‘self-eating', autophagy was first proposed by Christian de Duve at the 1963 Ciba Foundation Symposium on Lysosomes, after listening to a presentation on Alex Novikoff's recent work [1]. The previous decade, Sam L. Clark and Novikoff had observed mitochondria inside membrane-bound compartments with lysosomal enzymes in mouse kidneys [2, 3], representing the first image of what we now know as autophagy.

Autophagy involves an ensemble of cellular mechanisms, all of which promote the degradation of cytosolic components (macromolecules or organelles) by the lysosome [4, 5]. Three different types of autophagy are described in mammalian cells: macroautophagy, microautophagy, and chaperone-mediated autophagy (CMA). Only macroautophagy involves the participation of a double-membrane organelle called the autophagosome, which engulfs the cytosolic component (cargo) and delivers it to the lysosome for degradation [6]. In microautophagy, the cargo is directly taken up by the lysosome, where it is degraded through invagination of the lysosomal membrane [7]. CMA is a very specific type of autophagy described only in mammalian cells whereby cytosolic proteins are selectively identified by a chaperone protein that delivers them to the surface of the lysosome [8]. The chaperone interaction with the CMA receptor at the lysosomal membrane allows the receptor dimerization, and the unfolding and translocation of the protein inside the lysosome following degradation. This review will focus specifically on macroautophagy (henceforth referred to simply as “autophagy”) and its roles in mammalian neurodevelopment, neurogenesis, and associated diseases.

### Molecular mechanisms of autophagy

Autophagy and the components involved are highly conserved from yeast to mammals. At the molecular level, autophagy is orchestrated by a cohort of proteins generically referred to as autophagy-related (ATG) proteins. The majority of these proteins were first characterized in yeast, and their respective mammalian orthologues were subsequently described. To date, 42 ATG proteins have been described in yeast, most of which are conserved in mammalian cells, where they mediate different roles at discrete stages of the autophagy process [9, 10].

Macroautophagy differs from other forms of autophagy as it involves the autophagosome, a large double-membrane structure (mean diameter in mammalian cells, 0.5–1.5 µm) that is transiently formed upon autophagy induction. Despite intensive research, the precise origin of the autophagosome remains unclear. It is generally accepted that the autophagosome emerges from a pre-existing structure called the phagophore or isolation membrane (IM), but the source of the membrane that comprises the IM is still under debate. It appears that the IM originates from endoplasmic reticulum (ER), specifically from membrane compartments enriched in phosphatidylinositol 3-phosphate (PI3P), sometimes referred to as omegasomes [11]. However, other membrane sources also implicated in the autophagosome formation include the mitochondria, plasma membrane, lipid droplets, Golgi apparatus, and even endosomes. The current theory is that multiple membrane sources can supply lipids for autophagosomal membrane formation, a process in which ER-mitochondria contact sites appear to play an increasingly important role [12]. However, it is unclear how and when these different membrane compartments participate in autophagosome formation and whether this is stimulus- or even cell-type-dependent [13].

Regardless of the specific origin of the IM, initiation of autophagy and formation of the autophagosome involve the generation of two protein complexes. The first initiation complex, commonly known as the UNC51-like kinase-1 (ULK1) complex, comprises the following proteins: the *unc-51-*like autophagy activating kinase 1 (ULK1, mammalian orthologue of yeast Atg1); ATG13; ATG101; and focal adhesion kinase family interacting protein of 200 kD (FIP200, a mammalian orthologue of yeast Atg17). The ULK1 complex is assembled and activated at the IM, where it recruits and phosphorylates the transmembrane protein ATG9, which in turn is inserted into the IM [14]. Phosphorylation of ATG9 by the ULK1 complex triggers the recruitment of phospholipids from various sources, initiating elongation of the IM [15]. ULK1 also phosphorylates key components of the second key protein complex, the class III phosphatidylinositol 3-kinase (PI3K) complex, an action required for initiation of autophagy [16]. The PI3K complex comprises the following proteins: the PI3K catalytic subunit type 3 (PIK3C3, also known as vacuolar protein sorting 34 [VPS34]); PI3K regulatory subunit type 4 (PIK3KR4, also known as VPS15); ATG14; Beclin 1 (BECN1, mammalian orthologue of yeast Atg6); and the nuclear receptor binding factor 2 (NRBF2). The PI3K activity of this complex produces PI3P units, which also integrate into the IM and further contribute to its elongation [17]. Another critical event in autophagosome formation involves the activity of two ubiquitin-like conjugation systems. In the first of these systems, the E1-like activation enzyme ATG7 activates ATG12 and the E2-like conjugation enzyme ATG10 induces the formation of the ATG5-ATG12 conjugates. ATG5-ATG12 conjugates act as E3-like ligation enzymes, binding ATG16L to form the ATG5-ATG12-ATG16L complex, which in turn dimerizes and associates with the outer membrane of the nascent autophagosome [18]. In the other conjugation system, the cysteine protease ATG4 cleaves the microtubule-associated proteins 1 light chain 3 beta (MAP1LC3B, one of the mammalian orthologues of yeast Atg8 and commonly known as LC3). Cleaved LC3 (or LC3-I) is subsequently activated by ATG7 and lipidated by E2-like conjugation enzyme ATG3 and the ATG5-ATG12-ATG16L complex, which acts as an E3-like ligation enzyme [19]. Lipidated LC3, commonly referred to as LC3-II, is distributed on the outer and inner membranes of the forming autophagosome [20], contributing to its elongation and closure [21] while also recruiting autophagy receptors that bind the cargo targeted for degradation [16, 22].

Autophagy has long been viewed as a non-selective degradative mechanism. While in CMA the cargo is selected by chaperones with a high degree of specificity, the autophagosome can engulf a broad range of cargo types. This appears to contradict the selective nature of autophagy. However, recent evidence indicates that several molecules that are recruited by Atg8 to the forming autophagosome can act as autophagy receptors, providing a degree of specificity in terms of the cargo selected for degradation. These autophagy receptors, the list of which is continuously growing, use the LIR (LC3-interacting region) to act as a bridge between Atg8 family members and the cargo [23]. An excellent review of selective autophagy receptors can be found elsewhere [24]. The most studied autophagy receptors are p62 and NBR1, which participate in the degradation of protein aggregates (aggrephagy) and damaged organelles; NDP52 and TAX1BP1, which are implicated in autophagy of damaged mitochondria (mitophagy) and bacteria (xenophagy); and OPTN, which plays important roles in aggrephagy, mitophagy and xenophagy [24].

The last stages of the autophagy process involve closure of the IM, resulting in engulfment of the cargo, and fusion of the autophagosome with a lysosome to give rise to the autolysosome, in which the cargo is degraded [25]. The process of fusion and formation of the autolysosome involves various proteins of the endocytic pathway, including the Ras-related protein RAB7A, soluble N-ethylmaleimide-sensitive factor activating protein receptor (SNARE) proteins, and the lysosome-associated membrane protein 2 (LAMP2) [16]. Before fusing with the lysosome, the autophagosome can fuse with a late endosome resulting in the formation of an intermediate structure called amphisome, which in turn fuses with a lysosome to proceed with the degradation of the cargo [26]. In both cases, macromolecules and organelles are degraded due to the action of acidic hydrolases in the lysosomal lumen. Finally, as the process of cargo degradation is completed, the inner membrane of the autolysosome is disassembled and the resulting biomolecules are released and recycled in the cytosol.

### Regulation and function

Given its importance for cell homeostasis, it is not surprising that autophagy is tightly regulated. The induction of autophagy in mammalian cells is under the control of multiple signaling pathways, most of which converge on the mechanistic target of rapamycin (mTOR), a central regulator of multiple cellular processes, including cell growth and differentiation, that is localized in lysosomes [27]. Nutrient starvation is the best characterized autophagy-inducing stressor. Under nutrient rich conditions (resting state), initiation of autophagy is prevented by mTOR complex 1 (mTORC1), which inactivates the ULK1 complex. During starvation, the decrease in lysosome amino acid levels triggers mTOR release from lysosomes, resulting in reduced phosphorylation of its targets [28]. mTORC1 consists of the regulatory-associated protein of mTOR (RAPTOR), the proline-rich AKT1 substrate of 40 kDa (PRAS40), the mammalian lethal with SEC13 protein 8 (MLST8), and the DEP domain-containing mTOR-interacting protein (DEPTOR). Active mTORC1 inactivates ULK1 through phosphorylation of Ser757 [29], preventing initiation of autophagy. Conversely, decrease in adenosine triphosphate (ATP) results in increased AMP levels in the cell, which in turn promote autophagy via AMP activated protein kinase (AMPK), which inactivates mTORC1 [30].

The role of AMPK in promoting the initiation of autophagy goes beyond its inactivation of mTORC1. Recent evidence shows that AMPK activity phosphorylates TFEB and TFE3, two key transcription factors that regulate the expression of many autophagy and lysosomal proteins after their nuclear translocation [31]. Furthermore, under conditions of reduced ATP and increased AMP levels, active AMPK also promotes autophagy by activating ULK1 and class III PI3K complexes. AMPK directly activates ULK1 through phosphorylation of Ser317 and Ser777 [29], and the regulatory subunit BECN1 through phosphorylation of Ser91 and Ser94 [32]. The class III PI3K complex is also activated at the level of BECN1 by ULK1, which directly phosphorylates BECN1 at Ser14 [15], and by the activating molecule in BECN1-regulated autophagy (AMBRA1) [33]. By contrast, initiation of autophagy is prevented by the antiapoptotic protein BCL2, which binds to and inhibits BECN1 [34]. In summary, multiple signaling pathways regulate autophagy by interacting with different components, resulting in an intricate regulatory network that promotes or prevents the initiation of autophagy depending on cellular conditions.

The main result of autophagy is degradation of macromolecules and cellular organelles. Under resting conditions initiation of autophagy is generally blocked by mTORC1, whereas under low ATP conditions autophagy is promoted by AMPK. Typically, autophagy can be induced *in vitro* and *in vivo* by nutrient deprivation or starvation; the lack of any type of essential nutrient (e.g. glucose, amino acids) in growth medium effectively induces autophagy as the decrease in the metabolic rate reduces ATP levels [35]. Under these circumstances, the utility of autophagy lies in the reutilization of small biomolecules produced by degradation of macromolecules and organelles (e.g. amino acids resulting from protein degradation can be used as an energy source through the tricarboxylic acid cycle). However, it is important to note that autophagy also occurs constitutively (i.e. without nutrient deprivation) in all cells. Another important role of autophagy is to eliminate abnormal or excess cytosolic components in order to prevent their accumulation. In autophagy-deficient cells aberrant proteins and organelles accumulate in the absence of any external stressor [36]. Other recently proposed roles of autophagy not directly related to its degradative function include transport of proteins from the cytoplasm to the lysosome [37] and unconventional secretion of proteins that lack the typical signal peptide [38]. Many autophagy proteins have also non-autophagy-related functions that are often overlooked, and can confound the interpretation of their functions [4]. It is therefore advisable to validate phenotypes by knockdown of several autophagy regulators [39, 40].

### Autophagy and neuronal diseases

Autophagy has been implicated in multiple diseases, especially neurodegenerative and inflammatory diseases, autoimmune disorders, and cancer [41]. Research conducted over years has probed the connection between autophagy and neurodegeneration. Because the accumulation of unfolded or misfolded proteins and damaged organelles is a hallmark of most neurodegenerative disorders, the link between neurodegeneration and dysregulation of neuronal autophagy has been proposed [42].

Autophagy provides a mechanism by which neurons eliminate intracellular damaged components. Therefore, defective autophagy could reasonably result in the accumulation of altered proteins and organelles, leading to neurodegeneration. Over a decade ago, massive accumulation of proteins and progressive loss of neurons were described in mice in which the essential autophagy factors ATG5 [43] and ATG7 [44] were specifically absent in the central nervous system (CNS). Although these conditional knockout (KO) mice are born viable, they eventually die prematurely [43, 44]. However, analyses of the embryos revealed diffuse ubiquitinated proteins and inclusions in dorsal root ganglion neurons, indicating that alterations in proteostasis occur as early as embryonic development. Moreover, depletion of the essential autophagy factor FIP200 in mouse neuronal brain cells results in neuronal loss preceded by accumulation of ubiquitinated protein aggregates, damage to mitochondria, and axonal degeneration [45]. The results of these studies clearly indicate that autophagy is crucial to prevent neurodegeneration and that deficient autophagy may contribute to the development of neurodegenerative disorders, starting during development.

A second link is implied by the recently established role of autophagy in protein homeostasis and neuronal activity in the presynaptic terminal (see following section). In this context, deficits in autophagy would be expected to impair synaptic function, leading to synaptic loss and neurodegeneration. Several studies have shown that neuronal stimulation induces upregulation of autophagy and accumulation of autophagosomes in synaptic terminals and axons [46–49]. Additionally, the loss of neuronal autophagic function leads to abnormal accumulation of ER structures in the axon, resulting in elevated calcium release and abnormal synaptic neurotransmission [50]. These evidences highlight the importance of autophagy to maintain neuronal function in a healthy brain.

Multiple studies have shown that mutations in several autophagy-related genes are risk factors for human neurodegenerative disorders. For instance, the most common mutations associated with Parkinson's disease affect two proteins, PTEN-induced kinase 1 (PINK) and PARKIN, both of which are essential for mitophagy [51, 52]. In Alzheimer's disease, mutations in *PRESENELIN 1* result in increased lysosomal pH [53], while mutations in the amyloid precursor protein indirectly lead to RAB7A activation [54], in both cases resulting in impaired lysosomal activity and altered autophagic function [55]. Finally, mutations in genes that participate in the molecular mechanisms of autophagy, including *TBK1, VAPB, ATP13A2* and *SQSTM1* among others, are also implicated in neurodegenerative diseases [41].

## AUTOPHAGY IN NEURODEVELOPMENT AND NEUROGENESIS

### Autophagy and the neuron: a lifelong relationship

Neurons are highly specialized cells that form the nervous system. These post-mitotic cells cannot divide or be easily replaced, and are therefore particularly sensitive to any loss of homeostatic balance. It thus seems logical that autophagy may be even more essential for neurons than for other cells. As early as the 1960s, studies of the rat sciatic nerve revealed the presence of autophagosomes in neurons [56]. However, despite years of research, the role of autophagy in neurons is less well understood compared with other mammalian cells and tissues. One of the difficulties that arises when studying autophagy in neurons is that starvation, which is widely used to induce autophagy in several *in vivo* and *in vitro* models, does not clearly upregulate autophagy in either the mouse brain or primary cultures of mouse neurons. The first studies in mice revealed no detectable upregulation of autophagy in the brain after 48 hours of starvation, in contrast with other tissues such as liver, muscle, and heart, in which autophagy was upregulated [57]. However, subsequent studies using novel approaches demonstrated starvation-induced upregulation of autophagy in mouse cortical neurons and Purkinje cells [58]. Our group has also shown that food starvation in mice increases autophagic flux in the retina [59]. Nonetheless, starvation may not be the best inducer of autophagy in the brain given the brain's unique capacity to resist and adapt to nutrient deprivation. The insulin-glucagon hormonal system, which is modulated by the neurons of the hypothalamus, ensures that the brain receives an adequate supply of nutrients, for instance by inducing the catabolism of glycogen or adipose fat from peripheral sources. Starvation-dependent autophagy may also be regulated differently in distinct neuronal cell types and in glia [60]. A recent *in vitro* study reported that primary astrocytes are much more responsive to starvation than primary hippocampal neurons. Intriguingly, the study also showed that the buffer used in the cell culture media (bicarbonate versus HEPES) affected the response of astrocytes, but not primary neurons, to metabolic stress [61]. Further studies are needed to determine how neurons and glia activate autophagy in different settings and how their interactions modulate homeostatic mechanisms. Neurons (at least when studied *in situ* inside the brain) may not need to resort to upregulate autophagy under conditions of acute starvation, but this does not mean that autophagy is not important for neuronal function.

Other important pathways also involved in regulating neuronal and glial starvation-induced autophagy include the JNK/p38 pathway[62], calcium signalling [63], and the Hippo pathway [70]. JNK has been implicated in the phosphorylation of the Ser70, Ser87, and Thr69 residues of BCL2 [64], resulting in the dissociation of BCL2 and BECN1 and inducing autophagy under starvation conditions [34, 64, 65]. p38 also contributes to autophagy regulation: in response to lipopolysaccharide stimulation, p38α MAPK directly phosphorylates ULK1 in microglia, inhibiting ULK1 activity, disrupting its interaction with ATG13 in the autophagy initiation complex, and thereby reducing autophagic flux [66]. Ca^2+^ signalling also appears to influence autophagy regulation, as starvation induces lysosomal Ca^2+^ release and the formation of a lysosomal Ca^2+^ microdomain that activates calcineurin, resulting in subsequent activation of TFEB [67]. Ca^2+^ upregulation also regulates autophagy in the brain: CaMKK-b, a Ca^2+^-activated kinase, was recently identified as a direct activator of AMPK [63]. New data suggest that ER-located Bcl-2 inhibits autophagy induced by Ca^2+^ mobilizing agents by regulating Ca^2+^ homeostasis in a Beclin 1-independent manner [68]. Finally, the Hippo pathway, which primarily regulates tissue growth and organ size, is yet another signalling pathway implicated in autophagy regulation [69]. Glucose starvation increases AMPK-dependent phosphorylation of YAP [70], one of the main downstream transcription coactivators of the Hippo pathway. Moreover, recent findings have demonstrated a role of STK3/MST2 and STK4/MST1, the main components of the Hippo pathway, in the regulation of autophagy. Phosphorylation of LC3 at threonine 50 by these proteins is fundamental for autophagosome-lysosome fusion and subsequent elimination of the cargo [71].

Because neurons do not divide, as previously stated, they require strict quality control mechanisms to prevent the accumulation of damaged organelles and misfolded proteins. That basal autophagy is indeed an essential housekeeping mechanism is clearly demonstrated by the specific deletion of *Atg5* and *Atg7* in neuronal precursors, which results in death in three month old mice due to severe neurological defects including motor deficits and neurodegeneration [43, 44]. Moreover, the neurons of these animals accumulate polyubiquitinated proteins and p62, giving rise to inclusion bodies that become more numerous with increasing age. These mice also exhibit retinal alterations including photoreceptor death and night vision loss [72]. That neurons have a higher basal level of autophagic flux than other cell types is well accepted, and underscores the importance of autophagy for the maintenance of homeostatic balance and neuronal function [73, 74]. Neurons are polarized cells that in some cases have very long axons and neurites, and autophagosome formation and degradation has been shown to be differentially regulated in a compartment-specific manner, showing differential regulation in the soma vs. the axon for example [73]. How this correlates *in vivo* with neuronal function remains to be determined.

The many physiological alterations that accompany aging include macromolecular damage affecting all subcellular compartments. Lysosomal damage impairs the ability of aged tissues to eliminate damaged organelles and macromolecules [75]. In the CNS aging can also be accompanied by progressive mitochondrial dysfunction, resulting in chronic bioenergetic inefficiency and the generation of free radicals that damage cellular structures, including membranes [76, 77]. Although failure of one of the cell's catabolic systems (e.g. the ubiquitin proteasome system or autophagy) is generally compensated for by another, aging is accompanied by a generalized decrease in catabolism, exacerbating cell and tissue damage. Finally, defects in the autophagy-lysosomal system favour the accumulation of misfolded proteins that contribute to the development of diseases typically associated with aging such as Alzheimer's disease, Parkinson's disease, and cataracts [78].

### Autophagy in neural stem cell proliferation and differentiation

The processes of neuronal formation (neurogenesis) and differentiation (part of the broader process of neurodevelopment) have their own particularities and are especially important, as generally the mammalian brain produces very few novel neurons after development. During neurodevelopment, immature neurons are formed from neuronal stem cells (NSCs) and differentiate into mature, fully-functional neurons that are capable of establishing synaptic connections and producing excitatory or inhibitory action potentials, resulting in an intricate neuronal network that forms the nervous system. All these events are tightly regulated, as any failure in brain development can make the embryo unviable or cause mental retardation. Maintenance of homeostasis is therefore essential. Moreover, as described for other cell types and tissues, autophagy may play an essential role in neuronal formation and differentiation [79].

NSCs are multipotent cells that can self-renew and proliferate to produce progeny cells and terminally differentiate into neurons, a process commonly referred to as neurogenesis. In mammals, neurogenesis mainly occurs during embryonic development from NSCs located in the ventricular zone of the developing CNS [80]. However, it has been now accepted for years that neurogenesis is also present in the adult brain in the subgranular zone in the dentate gyrus of the hippocampus and in the subventricular zone of the lateral ventricles [81]. Like all stem cells, NSCs are maintained in a pluripotent state that allows them to divide continuously in order to self-renew and generate new neurons and glial cells. Accordingly, maintenance of homeostasis, elimination of protein aggregates, and degradation of organelles by autophagy are essential for NSC survival during development and adulthood [82].

**Figure 1:**
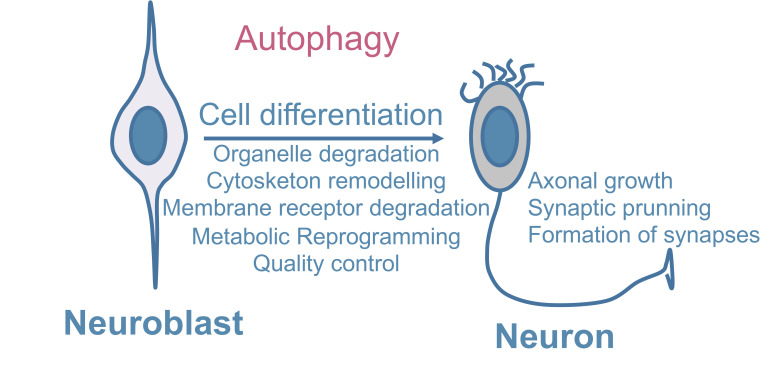
FIGURE 1: Main roles of autophagy for neuronal differentiation. During neurodevelopment, neural stem cells and neuroblasts proliferate and differentiate to give rise to postmitotic neurons. Autophagy has a crucial role in differentiation by promoting cell remodelling including cytoskeleton and membrane receptor degradation as well as organelle degradation. Other functions include metabolic reprogramming and quality control functions. Moreover, other processes that occur during development, such as axonal growth, formation of synapses and synaptic pruning are regulated by autophagy.

A study using *Atg16L1* hypomorph mice and primary neurons showed that NOTCH, a plasma membrane receptor and master regulator of neuronal development, is taken up into ATG16L1-positive autophagosome-precursor vesicles and modulates neurogenesis, indicating that neuronal differentiation can be controlled by the selective autophagic degradation of membrane receptors [83]. Thus, in addition to its classical quality-control function, autophagy plays a variety of other roles in controlling the maintenance and differentiation of NSCs (**Figure 1**) [84].

### Autophagy and metabolic control in neurodevelopment

Autophagy is an important regulatory component of metabolic function because it supplies metabolic substrates obtained by degradation of cytosolic macromolecules and organelles. In fact, autophagy is typically described as a compensatory metabolic mechanism, activated in response to starvation or nutrient deprivation. However, as mentioned before, this function of autophagy appears to be limited in the brain and during neurodevelopment.

Recent studies have indicated additional roles of autophagy in regulating the proliferation and differentiation of NSCs. For example, several findings suggest important roles of autophagy in sustaining metabolic reprogramming during stem cell differentiation [84]. Our group has demonstrated that the expression of *Atg7, Beclin1, LC3,* and *Ambra1* is markedly increased in cultured embryonic mouse olfactory bulb-derived NSCs during the initial period of neuronal differentiation [85]. In this setting *Ambra1* and *Atg5* deficiency decreases neurogenesis, a phenotype that can be reversed by methylpyruvate supplementation, suggesting that progenitor cells activate autophagy to meet their high energy demands [85].

Autophagy plays an additional metabolic role via the process of mitophagy [86]. Mitochondria, referred to as the “powerhouses of the cell”, are organelles that contain all the machinery required for oxidative phosphorylation and produce most of the cellular supply of ATP, the cell's main energy source [87]. Although mitophagy is commonly associated with the elimination of damaged or dysfunctional mitochondria in processes such as ageing and neurodegenerative disorders [88], recent evidence indicates a key role in neurodevelopment.

During neurodevelopment NSCs and immature postmitotic neurons undergo multiple changes. One such change is the neuron-glial switch, which is accompanied by increased levels of cell death, and another is a switch in their principal source of energy. Like all the other stem cell types, including cancer cells, NSCs and immature postmitotic neurons initially obtain the majority of their ATP via oxidative phosphorylation, which occurs in the mitochondria. After the aforementioned metabolic switch, glycolysis and lactate fermentation, which occur in the cytosol, become the main sources of energy. This phenomenon has been reported in a neuronal context in olfactory bulb NSCs [85] and in retinal ganglion cells [89]. Interestingly, studies conducted in retinal ganglion cells showed that the metabolic switch from oxidative phosphorylation to glycolysis and lactate fermentation is mediated by the selective elimination of mitochondria via mitophagy. Blockade of autophagy in general and mitophagy in particular not only prevents this metabolic reprogramming but also blocks neuronal differentiation of retinal ganglion cells [89]. This strongly suggests that autophagy participates in the metabolic switch that occurs during neurodevelopment by contributing to the elimination of mitochondria, the organelles responsible for oxidative phosphorylation.

What is the ultimate function of this metabolic reprogramming in neurodevelopment? It seems likely that the switch towards glycolytic and fermentative production of ATP is related to the proliferative capacity of NSCs. In fact, embryonic stem cells and hyperproliferative cancer cells generally depend on glycolytic metabolism rather than oxidative phosphorylation [90]. Proliferation (and subsequent neuronal differentiation) of NSCs entails high energy requirements and it may be more beneficial for NSCs to obtain the majority of this energy from glycolysis and lactate fermentation rather than from oxidative phosphorylation, even though the latter, which takes place in the mitochondria, is normally more efficient. This apparently paradoxical situation is comparable to the Warburg effect, which has been widely studied in cancer cells: these cells are also highly reliant on glycolysis for energy production, possibly because this route generates ATP faster than oxidative phosphorylation [91]. Hence, it is possible that NSCs also undergo a transient metabolic switch towards glycolysis and lactate fermentation during neurodevelopment in order to sustain their high level of protein synthesis for cytoplasm remodelling and axon growth, and that this switch is dependent on autophagy, which eliminates mitochondria and thereby prevents oxidative phosphorylation. Further research is required to confirm this hypothesis, especially *in vivo* studies demonstrating that mitophagy and metabolic reprogramming are indeed essential for proper mammalian neurodevelopment in other contexts besides the retina. In summary, while the metabolic transition from aerobic glycolysis to oxidative phosphorylation is regarded as a hallmark of neuronal differentiation, there are specific situations in which neuronal differentiation is controlled by the selective degradation of mitochondria via autophagy.

### Autophagy in axonal growth, synapse formation, and synaptic pruning

The complex morphology of neurons poses a unique challenge for proteostasis [73]. The results of genetic studies conducted in hypothalamic proopiomelanocortin (POMC) neurons suggest that autophagy is essential for axonal growth *in vivo* [92]. Loss of ATG7 in POMC neurons results in metabolic dysregulation caused by developmental defects in POMC neurons, including accumulation of ubiquitinated proteins and loss of axonal projections [92]. Moreover, *in vitro* studies have shown that mTOR signaling inhibitors and autophagy activators tuberous sclerosis complex 1 and 2 (TSC1 and TSC2) localize in axons in developing cultured neurons and are important for axonal growth [93]. Regardless, further *in vivo* studies of different brain areas are needed to confirm a specific requirement of autophagy for axonal growth.

*In vivo* studies of *Drosophila* larvae have shown that autophagy promotes synaptic formation and development at neuromuscular junctions [94]. Specifically, autophagy reduces the levels of Highwire, an E3 ubiquitin ligase that prevents overgrowth of neuromuscular junctions. A growing body of evidence also suggests that proper autophagy levels are key for neuronal plasticity, which is essential for the formation and elimination of synapses during neurodevelopment [95]. ATG5-deficient mice display increased excitatory neurotransmission in CA3-CA1 synapses as early as two months of age. This increase is due to the accumulation of tubular ER, which is caused by the abnormal autophagic function in the absence of ATG5 and results in increased calcium release from the ER lumen. Thus, neuronal autophagy is essential to regulate synaptic plasticity via control of calcium efflux from ER stores [50, 96].

Furthermore, autophagy may play a role in the elimination of synapses, a process of critical importance during neurodevelopment commonly referred to as synaptic pruning. This consists of the elimination of redundant, unnecessary, or inappropriate synapses that form during the early stages of neurodevelopment [97]. *In vitro* studies of ATG7-deficient hippocampal neurons have shown that these neurons are capable of developing dendritic spines and forming normal synapses, but are unable to subsequently prune the same number of spines as wild type neurons [98]. It will be essential to explore this phenomenon in mammalian models to confirm the requirement of autophagy for both the formation and elimination of synapses during neurodevelopment. Alterations in synaptic pruning are an underlying cause of multiple neurodevelopmental disorders. Interestingly, post-mortem analysis of brains from autism spectrum disorder (ASD) patients has shown that reduced synaptic pruning in certain brain regions is inversely associated with the levels of the autophagy marker LC3-II [98]. In the following section, we will discuss more thoroughly the association between autophagy and neurodevelopmental disorders.

## AUTOPHAGY AND NEURODEVELOPMENTAL DISORDERS

Regardless of existing knowledge gaps, available evidence highlights the importance of autophagy in neurodevelopmental processes. Therefore, deficits in autophagy could feasibly lead to defective neurodevelopment and the appearance of neurodevelopmental disorders. Indeed, evidence published in recent years points to an important role of autophagy in several neurodevelopmental disorders.

Distinguishing neurodevelopmental from neurodegenerative diseases poses a challenge: both concepts imply neuronal demise, albeit over different time frames. According to the fifth edition of the Diagnostic and Statistical Manual of Mental Disorders, the term “neurodevelopmental disorders” describes a group of conditions with onset in the developmental period, usually before five years of age, that cause impairment of personal, social, academic, or occupational functioning, ranging from specific limitations (e.g. motor control deficits) to global impairment (e.g. deficits in social skills or intelligence) [99]. Mutations in a variety of genes related directly or indirectly to the autophagic process have been identified in different neurodevelopmental disorders (**Table 1**). In our discussion below on the consequences of autophagic dysfunction during neurodevelopment, neurodevelopmental disorders are grouped according to the stage of autophagy affected by the mutation and the clinical similarities. In broad terms, we can distinguish between neurodevelopmental disorders involving mTOR hyperactivity and those in which autophagy genes are dysregulated.

**TABLE 1.** Main neurodevelopmental disorders where mutations in autophagy proteins impacts disease.

**Disease**	**Gene**	**Role in autophagy pathway**	**Main phenotype**	**References**
Tuberous Sclerosis Complex	TSC1/2 complex	mTOR Inhibitor	Non malignant tumours, epilepsy, autism	[95, 96]
Fragile X Syndrome	Fmr1	mTOR Inhibitor	Intellectual disability, autism, seizures, hypersensitivity, attention deficit, hyperactivity, growth and craniofacial abnormalitites	[97, 98]
Neurofibromatosis	NF1	mTOR Inhibitor	Café'au lait spots, bening and malignant tumours, epilepsy (10%), autism	[91, 99]
Lhermitte-Duclos Disease	PTEN	mTOR Inhibitor	Gangliocytomas in cerebellum, ataxia, seizures, autism (with macrocephaly)	[100]
TBCK Encephaloneuropathy	TBCK	mTOR activator	Intellectual disability, coarse face, hypotonia, leukoencephalopathy, neuronopathy, seizures	[169]
BPAN	WDR45 (WIPI4)	Autophagosome formation and elongation Autophagosome-lysosome fusion	Iron accumulation in basal ganglia 1^st^ phase (neurodevelopmental): global developmental delag, intellectual disability, psychomotor retardation, febrile seizures, autistic features 2^nd^ phase (neurodegenerative): cognitive decline, dementia, dystonia, parkinsonism, optic nerve atrophy	[122,125, 127]
Intellectual disability associated to WIPI3	WDR45b (WIPI3)	Autophagosome formation and elongation Autophagosome-lysosome fusion	Intellectual disability	[123]
Vici Syndrome	EPG5	Autophagosome-lysosome fusion	Corpus Callosum agenesis, cataracts, cardiomyopathy, immunodeficiency, hypopigmentation, muscle and neurogenic anomalies, epilepsy, microcephaly and loss of learned skills	[122, 154–156]
Spastic Paraplegia 11	ZFVE26 (coding for spatacsin)	Lysosome Biogenesis	Spasticity, pyramidal weakness, corpus callosum affection, ataxia (less common)	[136,138, 139]
Spastic Paraplegia 15	KIAA1840 (coding for spastizin)	Autophagosome maturation Lysosome Biogenesis	Spasticity, pyramidal weakness, corpus callosum affection, ataxia (less common)	[136, 138, 139]
Spastic Paraplegia 49	TECPR2	Autofagosome formation (functional endoplasmic exit sites) Autophagosome-lysosome fusion	Spasticity, pyramidal weakness, corpus callosum affection	[147]
SNX14-associated autosomal recessive cerebellar ataxia	SNX14	Autophagosome-lysosome fusion	Poor coordination (ataxia), cerebellum atrophy and Purkinje cell loss, mental retardation, seizures	[122, 166, 167]
Ataxia related to Atg5	Atg5	Autophagosome elongation	Ataxia, mental Retardation, development delay	[170, 174]

### Neurodevelopmental disorders associated with mTOR hyperactivity

**Figure 2:**
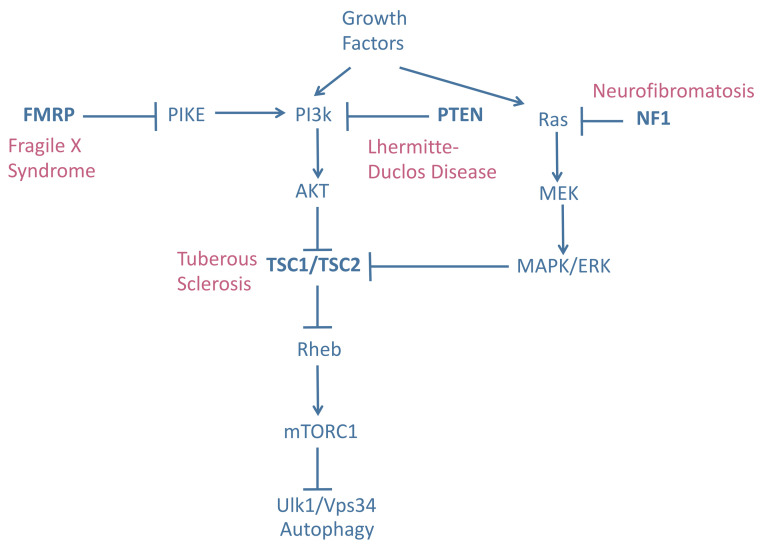
FIGURE 2: Upstream mTOR regulation and associated mutations causing mTORpathies. The TSC1/TSC2 complex, FMRP, PTEN and NF1 are negative regulators of mTORC1. Loss of function-mutations in these genes lead to an increased mTORC1 activity and a reduced autophagy induction and produce a subset of neurodevelopmental disorders known as mTORopathies. TSC1/2: Tuberous sclerosis complex 1/2, FMRP: Fragile X mental retardation protein, PTEN: Phosphatase and tensin homolog deleted on chromosome ten and NF1: Neurofibromatosis type 1.

mTOR is one of the central suppressors of autophagy. Hyperactivation of mTOR therefore decreases autophagic flux. If this effect is prolonged it can have detrimental effects on cell physiology. Mutations in the mTOR inhibitor genes tuberous sclerosis complex 1/2 (*TSC1*/*TSC2*), neurofibromatosis type 1 (*NF1*), phosphatase and tensin homolog deleted on chromosome ten (*PTEN*), and fragile X mental retardation 1 (*FMR1*) lead to tuberous sclerosis complex (TSC), neurofibromatosis, Lhermitte-Duclos disease (LDD), and Fragile X syndrome (FXS), respectively [100, 101], **Figure 2**. These genes negatively regulate mTOR activity in different ways. TSC1/2 acts as a GTPase-activating protein, deactivating Rheb-GTP by converting it into Rheb-GDP and thereby preventing the activation of mTOR kinase activity by Rheb-GTP [102]. NF1 supresses Ras, an upstream regulator of ERK signaling and, consequently, of the TSC1/2 complex [103]. Finally, PTEN and FMR1 regulate mTOR activity through PI3/Akt signaling [104] (**Figure 2**). These disorders are known as mTORopathies as their common biochemical feature is mTOR hyperactivity.

TSC manifests clinically with the appearance of benign tumours in multiple organs and is caused by mutations affecting the activity of the TSC1/2 complex [105]. Neural disorders are common in TSC patients: 90% have epilepsy and 50% have ASD [106]. FXS patients are severely intellectually disabled and are usually autistic, making FXS the most common inherited cause of ASD. Other characteristic clinical signs of FXS include seizures, hypersensitivity, attention deficit, hyperactivity, and growth and craniofacial abnormalities [107]. FXS is caused by the expansion of a poly-CGG trinucleotide repeat in the 5'UTR of the *FMR1* gene, which causes gene silencing and reduced expression of fragile X mental retardation protein (FMRP) [108]. Neurofibromatosis is a neurocutaneous disorder characterized by *café-au-lait* spots and benign and malignant tumours, with variable organ involvement. Epilepsy affects 10% of neurofibromatosis patients and ASD is a common manifestation of the disease [101, 109]. Finally, LDD is caused by mutations in *PTEN* and is part of a group of disorders known as PTEN hamartoma tumour syndrome. LDD is characterized by the presence of gangliocytomas in the cerebellum, ataxia, and seizures [101]. *PTEN* mutations have also been identified as a cause of ASD with macrocephaly [110]. In summary, mTORopathies are a group of genetic diseases that confer a high degree of susceptibility to ASD and epilepsy [100, 101], highlighting the importance of mTOR in epileptogenesis and the development of autism.

ASD is one of the most important neurodevelopmental disorders worldwide. It is characterized by restricted and repetitive behaviour and impairment of social interactions and verbal and non-verbal communication [111]. Autism is a multi-factorial disorder resulting from the interaction between genetic and non-genetic risk factors. Genes linked with the development of autism include mTOR genes as well as those implicated in modulating the balance between excitatory and inhibitory neurotransmission, cerebral connections, and synaptic plasticity [112]. These are all events in which autophagy may play a major role, as described previously. Epilepsy is a common neurological disorder that affects 1% of the world's population [101], and is characterized by spontaneous bursts of neuronal activity resulting in seizures [113]. Epileptogenesis, the process by which the brain becomes epileptic, can be the result of various causes, including structural brain lesions, congenital malformations, alterations in neuronal signaling, and defects in neuronal maturation and plasticity. In the neurodevelopmental context, the most common causes of epileptogenesis are malformations of the cortical layer and a reduction in the arrival of inhibitory interneurons to the cortical plate [113].

mTOR appears to play an important role in ASD and epileptogenesis. Although one of the key suppressors of autophagy, mTOR also participates in other processes (e.g. microtubule organization and protein synthesis [114]) that may be implicated in the autistic and epileptic phenotype. Studies performed over the last decade have reinforced the link between impaired autophagy and these disorders. A study of human post-mortem temporal lobe tissue from autistic patients revealed increased dendritic spine density and reduced developmental synaptic pruning associated with mTOR hyperactivation and impaired autophagy, as well as low levels of LC3-II and high levels of p62 [98]. These results indicate a strong correlation between mTOR hyperactivation (and therefore autophagy dysfunction) and defective synaptic pruning in autistic patients, but do not confirm a key role of autophagy in this process.

Animal models research strongly supports a central role of autophagy in social interactions. *Pten* mutant mice show impaired social behaviour and abnormal neuronal arborization, a phenotype that can be prevented by treatment with the mTOR inhibitor rapamycin [115]. A similar study of *Tsc2*^+/-^ mice showed that rapamycin treatment corrects ASD-like behaviours and ameliorates spine-pruning defects, but does not rescue the phenotype in double mutant *Tsc2*^+/-^:*Atg7*^CKO^ mice, supporting the view that autophagy is necessary to reverse the pathology [98]. Mice with *Atg7*-deficient microglia also show impaired synaptic pruning, abnormal social interactions, and repetitive behaviours [116], highlighting the importance of this process in a specific non-neuronal cell type. However, the potential role of synaptic pruning in the development of autism should be considered with caution. Hui *et al.* recently reported that *Atg7* deletion in GABAergic interneurons and excitatory neurons of the medial prefrontal cortex of adolescent mice, after completion of synaptic refinement, causes behavioural abnormalities including reduced social interaction, altered nesting behaviour, and increased anxiety, suggesting that autophagy continues to play a role in social behaviour after postnatal development. This observation points out to the idea that behavioural deficits arising from autophagy deficiency may not be related to neurodevelopment and synaptic pruning, but to the disruption of neurotransmission processes independent of age [117]. The authors also described a novel pathogenic mechanism based on defective autophagy-dependent neurotransmission whereby suppression of autophagy, either due to mTOR hyperactivation or mutation of autophagy genes, disrupts GABA_A_ receptor trafficking via sequestration of GABARAPs by p62-positive aggregates. The decrease in GABA_A_ receptor expression results in a reduction of inhibitory input and consequent neuronal hyperexcitability [117]. Autophagy therefore constitutes a link between mTOR hyperactivation, the neuronal excitation/inhibition balance, and seizures and ASD-like behaviour [118].

This mechanism proposed by Hui *et al.* is reinforced by previous research. The WDFY3 gene (or ALFY), which participates in the clearance of p62-positive aggregates [119, 120], has been detected as an ASD risk gene [121, 122]. The mechanism is also in agreement with findings in a mouse model of TSC, in which loss of TSC1 in pyramidal neurons leads to deficits in inhibitory synaptic function [123], possibly due to reduced GABA_A_ membrane translocation. Further evidence supporting a key role of GABA neurotransmission in TSC patients comes from the observation that TSC-related infantile spasms show a rapid and sustained response to treatment with vigabatrin [124], an irreversible inhibitor of the GABA degrading enzyme GABA transaminase. Finally, analysis of tissues from TSC patients has demonstrated reduced cortical expression of the GABA_A_ receptor subunit [125]. Reduced efficiency of GABAergic neurotransmission has also been described in mouse models of FXS [126], which show reduced mRNA expression of GAD67, the main GABA synthesizing enzyme [127]. However, while GABAergic transmission appears to be altered, it remains to be determined whether autophagy impairment is causally linked to this defect in FSX. In conclusion, it is tempting to speculate that decreased GABA_A_ membrane translocation may underlie the imbalance of excitatory and inhibitory synapses, and hence epilepsy and ASD, in patients with mTORopathies. However, the precise role of synaptic pruning in these pathologies remains to be elucidated.

### Neurodevelopmental disorders associated with impaired autophagy

**Figure 3:**
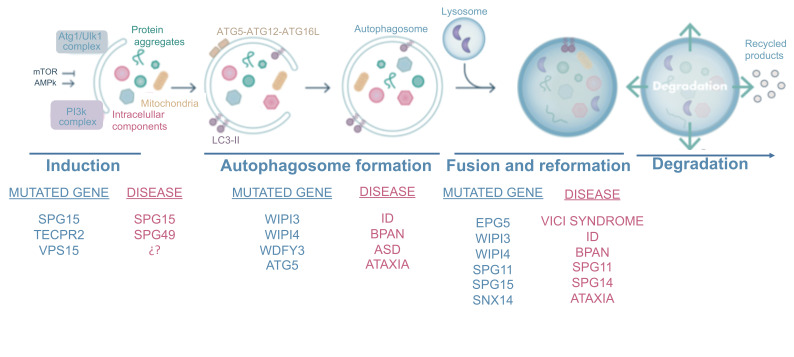
FIGURE 3: The autophagy process and most common mutated proteins in neurodevelopmental disorders. Autophagy is a highly conserved process that consists on the formation of a double membrane-vesicle engulfing cytosolic material and organelles, which finally fuses with lysosomes, where the cargo is degraded. ULK1 and PI3K complex participate in the autophagy induction, when the isolation membrane is formed. This membrane is elongated and specific materials are recognized by selective autophagic receptors until the closing and formation of autophagosome. The next step consists on the fusion with lysosomes and/or late endosomes, where cargo degradation takes place by lysosomal hydrolases. Finally, the resulting products are recycled and lysosomes can be reused through the process of lysosome reformation. Neurodevelopmental disorders described with mutations in autophagic genes are compiled according to the autophagic step affected. Defects in *WIPI3* and *WIPI4* have been found to correlate with ID and BPAN, respectively. The selective autophagic receptor WDFY3 gene, which participates in aggregates removal, is related with high susceptibility of ASD. Rare mutations have been found in the induction gene *VPS15* and autophagosome elongating gene *ATG5* in ataxic patients. Mutations in *SPG11, SPG15* and *TECPR2* produce hereditary spastic paraplegias. Finally, the *EPG5* and *SNX14* genes participating in the autophagosome/lysosome fusion are responsible for the Vici syndrome and ataxic disorders.

mTORopathies are not the sole cause of neurodevelopmental defects, which can also be caused by mutations in several genes involved in autophagy. These can be divided into disorders resulting from mutations affecting the early stages of autophagy, such as neurodegeneration with brain iron accumulation and hereditary spastic paraplegia 49 (SPG49), and those caused by mutations affecting later stages of the autophagy pathway, including hereditary spastic paraplegias 11 (SPG11) and 15 (SPG15), Vici Syndrome, and SNX14-associated ataxia. Here we describe the main phenotypes associated with these mutations (**Figure 3**).

#### β-Propeller protein-associated neurodegeneration (BPAN) and intellectual disability (ID)

Neurodegeneration with brain iron accumulation is a heterogeneous group of genetic disorders characterized by altered iron metabolism and iron accumulation in the basal ganglia [128], prominent extrapyramidal movement disorder, and intellectual deterioration [129]. Six different subtypes are described, depending on the gene affected [130], but here we will focus primarily on the subtype caused by mutations in *WDR45*, known as BPAN or static encephalopathy of childhood with neurodegeneration in adulthood. The course of BPAN has two distinct phases, beginning with ID, psychomotor retardation, febrile seizures, and even autistic features in childhood [129–131]. These clinical signs remain relatively stable until the second phase of the disease, in which progressive cognitive decline, dementia, dystonia, and parkinsonism are the predominant features [132]. It should be noted that mutations in *WDR45B*, a gene that shares a high level of homology with *WDR45*, are also associated with ID phenotypes [133].

*WDR45* and *WDR45B* encode the proteins WDR45 and WDR45B (also known as WIPI4 and WIPI3, respectively), which belong to the PROPPINs (β-propellers that bind polyphosphoinositides) family, a group of proteins that bind phosphatidylinositol 3,5-bisphosphate and PI3P via a phosphoinositide-binding motif [134, 135]. WIPI4 binds to the autophagic protein ATG2 and WIPI3 to FIP200, and both participate in the early stages of autophagosome formation [135, 136], probably acting upstream of PI3P production and downstream of LC3 to control the size of the nascent autophagosome [135]. WIPI3 and WIPI4 may participate in autophagosome-lysosome fusion by promoting lysosomal localization of the protein EPG5 [137], which is mutated in Vici syndrome, as discussed later in this review.

Depletion of WIPI4 in U2OS cells results in an increase in autophagosome number, without blocking autophagic flux [138]. Studies of lymphoblasts from BPAN patients indicate partial blockade of autophagic flux [129]. While ATG9A is found only transiently in the autophagosome under normal conditions, these lymphoblasts also contain enlarged structures positive for ATG9A and LC3, indicating improper autophagosome formation [129]. This observation suggests that the absence of WIPI4 affects autophagosome maturation, resulting in autophagosome accumulation before fusion with the lysosomal machinery. Primary cells from CNS-specific *Wdr45* KO mice also show defects in autophagic flux and the accumulation of protein aggregates. These mice partially recapitulate the BPAN phenotype, exhibiting extensive axon swelling, impaired motor coordination, and deficits in cognitive function [139]. *Wdr45b* KO mice present a more marked phenotype, characterized by severe motor deficits not present in *Wdr45* KO mice, including limb-clasping reflexes, ataxia, and much more severe neural defects [140]. Double KO (*Wdr45b*^*-/-*^; *Wdr45*^*-/Y*^) mice exhibit the same suckling defect seen in *Atg5* and *Atg7* KO mice, and more severe autophagy defects than single KO mice [140], confirming the role of both proteins in autophagy.

A key question is whether WIPI4 mutations cause global attenuation of autophagy or if they only affect a subset of selective autophagy. Recent studies in *Wdr45* KO mice and cells support a role of ER stress and the unfolded protein response in BPAN [141, 142]. On the other hand, defects in ferritinophagy could also explain the toxic accumulation of iron. Ferritinophagy is the selective autophagic degradation of ferritin, the main iron storage protein in cells [143]. Whether the absence of WIPI4 results in altered iron homeostasis due to defective ferritinophagy will need to be further investigated: while ferritin levels in fibroblasts from patients with WIPI4 mutations are reduced compared with controls, no such difference has been observed in neurons [144]. WIPI4 and WIP3 are therefore proteins participating in autophagosome formation and in the fusion step and mutations of them are related with two neurodevelopmental affections, BPAN and ID (**Figure 3**).

#### Hereditary spastic paraplegia (HSP)

HSP is a group of heterogeneous neurological disorders characterized by upper motor neuron degeneration resulting in spasticity of the lower limbs, spastic gait, and pyramidal weakness. Complex forms of this disease include additional neurological features such as epilepsy, ataxia, mental retardation, and extraneurological signs including cataracts, retinal degeneration, and skeletal abnormalities [145], and in some cases CNS structural abnormalities including thin corpus callosum (the connection between both hemispheres), white matter hyperintensities (as observed by magnetic resonance imaging), and cortical and cerebellar atrophy [145]. More than 50 spastic paraplegia genes have been identified to date [132]. Here, we discuss the three forms in which autophagy impairment has been described: HSP caused by mutations in *SPG11, SPG15*, and *SPG49*. Almost one third of complex HSP cases involve alterations in the corpus callosum, and 70% of these are due to mutations in *SPG15/ZFYVE26* or *SPG11/KIAA1840* [146]. *SPG11* and *SPG15* encode SPATACSIN and SPASTIZIN, respectively. SPASTIZIN is a large protein that participates in autophagosome maturation through interaction with the RUBICON-UVRAG-BECN1 complex. Cells without SPASTIZIN show reduced colocalization of LAMP1 and LC3, although autophagic flux is not completely blocked, as evidenced by increases in LC3-II levels following lysosomal inhibition with bafilomycin A1 [147]. It has been proposed that SPATACSIN and SPASTIZIN may participate in autophagic lysosome reformation, the process of lysosome biogenesis by which lysosomes are generated from pre-existing autolysosomes [148, 149]. In fact, recent research in mice points toward phosphatidylinositol 4-kinase type 2 alpha accumulation to be responsible for autophagic lysosome reformation disturbance in mice models of the disease [150]. In zebrafish, knockdown of spatacsin and spastizin results in motor impairment and defective branching of spinal cord motoneurons at neuromuscular junctions [151]. While no structural alterations have been reported in the brains of *Spg15* knockout mice, these animals develop a progressive spastic and ataxic gait disorder, and show neuronal loss in the motor cortex and cerebellum, resembling human HSP [152]. Before degeneration, these neurons accumulate large deposits of autofluorescent particles that colocalize with the lysosomal marker LAMP1, suggesting a link between lysosomal impairment and neurodegeneration [152]. Two different *Spg11* KO mouse models have been developed. The first was created by inserting a genetrap cassette into the first intron of the *SPG11* gene, resulting in the absence of SPATACSIN and consequently impairment of autolysosome reformation. This model reproduces the neurodegeneration in the motor cortex and cerebellum observed in SPG11 patients, but shows no early motor, anatomical, or cognitive deficits [153]. The second model, generated by Branchu *et al.* [154], simulates the most typical mutation found in SPG11 patients, and presents early-onset motor and cognitive deficits as well as lipid accumulation in lysosomes, as previously reported in SPG11 patients [155].

In 2012 Oz-Levi *et al.* identified a new form of complex HSP (SPG49) characterized by a thin corpus callosum and cerebral and cerebellar atrophy [156]. Patients with this complex form carry mutations in *TECPR2*, which encodes a protein that interacts with the six human Atg8 homologs and is a positive regulator of autophagosome accumulation [138]. TECPR2 regulates autophagy by maintaining functional endoplasmic exit sites, which serve as scaffolds for autophagosome formation [157]. However, recent studies also suggest a role for TECPR2 in the lysosomal targeting of autophagosomes [158, 159].

Other forms of HSP that involve dysregulation of lysosomal activity, and therefore of autophagy, are those caused by mutations in AP-5 subunits, including SPG48 (AP-5 ζ), and in AP-4 subunits, including SPG47 (AP-4 β1), SPG50 (AP-4 µ1), SPG51 (AP-4 σ1), and SPG52 (AP-4 σ1) [160]. AP-5 is the most recently identified of the five adaptor complexes and has been proposed to form a complex with SPATACSIN and SPASTIZIN that participates in protein sorting [161]. AP-4 is thought to participate in autophagosome biogenesis by regulating ATG9 export to autophagosomes [162]. These findings support the view that alterations throughout the entire autophagic pathway are implicated in corticospinal tract degeneration and the development of HSPs, as these disorders can be caused by mutations in genes involved in different stages of the autophagy process (**Figure 3**).

#### Vici Syndrome

Vici syndrome is a multisystem disease first described by Carlo Dionisi Vici in 1988 [163]. This hereditary disease is classically defined based on the presence of five characteristic features: agenesis of the corpus callosum; cataracts; cardiomyopathy; immunodeficiency; and hypopigmentation [132]. Muscle and neurogenic anomalies were later described in Vici syndrome patients [164–166], and in the last decade recessive mutations in *EPG5* were identified as cause of the disease [167]. Vici syndrome is a severe disease: most patients present developmental delay and die before the age of three [168]. *EPG5* is the homolog of the *Caenorhabditis elegans* gene *epg-5*, a specific autophagy gene that promotes fusion of autophagosomes with late endosomes/lysosomes during autophagy [169, 170]. Analysis of fibroblasts from Vici syndrome patients has revealed the accumulation of p62 and NBR1, levels of which do not increase in response to treatment with rapamycin and bafilomycin, suggesting impairment of autophagosomal cargo clearance in these cells [167]. However, colocalization of LC3 with LAMP1 is decreased in these fibroblasts, suggesting dysfunctional autophagosome-lysosome fusion. *Epg5*^*-/-*^ mice also show blockade of autophagic flux as a consequence of impaired proteolytic activity of autolysosomes [171]. A phenotype common to Vici syndrome patients and *Epg5*^*-/-*^ mice is agenesis of the corpus callosum, although the mouse model only partially reproduces the clinical features of the human syndrome [168]. Specifically, *Epg5*^*-/-*^ mice present selective degeneration of cortical layer 5 pyramidal neurons and spinal cord motor neurons, features reminiscent of the key clinical signs of amyotrophic lateral sclerosis [171]. This mouse also shows a decrease in the thickness of the outer nuclear layer of the retina from six months of age, and consequent reduction in electroretinogram responses, suggesting that it may constitute a useful model of retinitis pigmentosa [172]. Vici syndrome patients develop cataracts, and while retinal degeneration cannot be ruled out it is difficult to demonstrate owing to the short lifespan of these patients.

Vici syndrome is another example of a pathology caused by disturbed embryonic CNS development and aberrant autophagy. It features anatomical defects including callosal agenesis, which entails the ablation of white matter connecting the two brain hemispheres, and abnormal neuronal migration [167], in addition to behavioural and physiological defects. In fact, around two-thirds of Vici syndrome patients present severe epilepsy with different types of seizures, suggesting the inclusion of the syndrome in the context of mTOR-related disorders [166]. In addition to the neurodevelopmental features, loss of learned skills and microcephaly in children with Vici syndrome also suggest a neurodegenerative component [132]. This neurodegeneration has also been described in *Drosophila* following downregulation of *epg5* in the adult stage [166]. The neurodegeneration also observed in BPAN further reinforces the aforementioned functional connection between WIPI3, WIPI4, and EPG5.

#### SNX14-associated autosomal-recessive cerebellar ataxia

Hereditary cerebellar ataxia is a heterogeneous group of clinical conditions mainly characterized by imbalance and poor coordination with frequent cerebellar atrophy and Purkinje cell loss [173]. Two separate research groups recently identified mutations in the sorting nexin gene *SNX14* as the cause of a specific subtype of cerebellar ataxia known as autosomal recessive spinocerebellar ataxia 20 (SCAR20) [174, 175]. This clinical syndrome, which accounts for around 10% of early onset cerebellar atrophy and ataxia cases, is characterized by delayed development, cerebellar atrophy, motor ataxia, mental retardation and seizures [132, 176]. Some degree of overlap with lysosomal storage diseases is observed in certain cases [132], suggesting that cerebellar cells are highly sensitive to lysosomal impairment [174]. Sorting nexin (SNX) proteins are a large family of proteins characterized by the presence of a PX domain, which binds membrane phosphatidylinositol phospholipids, and a GTPase activating domain. Most SNX proteins (except SNX19) use this GTPase activating domain to regulate the G-protein signaling domain, thereby playing an important role in decreasing G-protein-coupled receptor signaling [177]. SNX14 is thought to bind to PI-enriched autophagosomes and mediate fusion with lysosomes, although this is yet to be definitively demonstrated [132, 177]. Animal models reproduce key features of SCAR20. Knockdown of SNX14 in zebrafish results in loss of cerebellar parenchyma and decreases in Purkinje cell number, autophagosome accumulation, and apoptosis [174]. Studies in mice suggest an important role of SNX14 in mouse neuronal development, as it is expressed at high levels in the brain, testes, and lungs and its expression increases further during development and maturation [178]. In fact, mice with Nestin-Cre-mediated *Snx14* deletion in neurons and glia recapitulate the pathological features of SCAR20 and present Purkinje cell loss and cerebellar degeneration [176]. *Snx14* knockdown in mouse cortical neurons also reduces intrinsic neuronal excitability, which correlates with decreased input resistance, underscoring the importance of SNX14 in neuronal excitability and synaptic function [178].

Cells from patients with *SNX14* mutations contain large lysosomes suggestive of lysosomal alterations and show impaired autophagosome clearance [174, 175]. These findings support a central role of lysosomal function in Purkinje cell viability and, therefore, the onset of cerebellar ataxia.

## FUTURE PERSPECTIVES

In this review we have summarized the key findings indicating a connection between autophagy, neurodevelopment, and neurodevelopmental disorders. Despite clear gaps in our knowledge of the relationships between these processes, the available evidence strongly supports a central role of autophagy in multiple neurodevelopmental processes.

The contribution of autophagy to neurodevelopment has been investigated both *in vitro* and *in vivo* in a range of animal models, the most common of which are *Drosophila* and mouse or rat models in which autophagy is either impaired by deletion of one or more autophagy-related genes or is directly inhibited using pharmacological approaches. A better understanding of the role of autophagy in neurodevelopment will require *in vivo* verification of *in vitro* findings and replication of the results obtained in mouse models through deletion of different autophagy-related genes. Several discrepancies arise between studies, depending on the experimental approach.

Mutations in genes that participate in a given stage of the autophagy pathway do not always give rise to the same neurologic phenotype, likely because the affected proteins can interact with different counterparts and participate in distinct cellular processes that influence the final phenotype. It is also possible that a given affected protein may play multiple, as-yet-unknown roles in the autophagy process. For example, WIPI4 and SPATACSIN both participate in early and late stages of autophagy. In addressing whether the impacted stage of autophagy can determine the neurodevelopmental disorder, some general conclusions can be drawn based on analyses of different disorders. First, it is important to note that epilepsy and seizures are features of almost every neurodevelopmental disorder described in this review (with the exception of HSPs), regardless of the stage of autophagy affected. This supports a role of autophagy in modulating the balance between excitatory and inhibitory synaptic connections. In fact, uncontrolled induction of autophagy also produces an epileptic phenotype: loss-of-function mutations in the mTOR activator gene *TBCK* give rise to a neurodevelopmental condition with ID, coarse facial features, hypotonia, leukoencephalopathy, neuronopathy, and seizures [179]. Therefore, epileptogenesis is described in patients with both impaired and hyperactivated autophagy. Second, autistic features are correlated with mTOR upregulation and defects in the early stages of the autophagy pathway, and are very common in mTORopathies. Finally, corpus callosum involvement and cerebellar atrophy appear to be more common consequences of defects in the later stages of autophagy. Alterations in the corpus callosum are observed in Vici syndrome and SPG patients, while cerebellar involvement is mainly observed in SCAR20. However, it is important to note that mutations in the gene encoding ATG5, which participates in the autophagosome elongation, have also been detected in ataxic patients [180], and that a mutation in VPS15 has also been reported in a child with different neurodevelopmental features: cortical and optic nerve atrophy, intellectual impairment, spasticity, ataxia, psychomotor affection and epilepsy [181]. These last two mutations are highly less frequent, but increment the complexity of our current understanding of neurodevelopmental disorders.

In this review we have proposed some correlations between alterations in specific stages of autophagy and discrete neurodevelopmental disorders. However, in order to acquire a more complete perspective it will be essential to further investigate how autophagy is differentially regulated in selected neuronal types, as this could provide crucial insights into the aetiology and pathogenesis of different neurodevelopmental disorders. Finally, it will be important to breach the species barrier and investigate the role of autophagy in human neurodevelopment. Human cerebral organoids and humanized brains or chimeric brains constitute valuable and valid models in which the contributions of autophagy to human neurodevelopment can be studied [182, 183].

## Abbreviatons:

AMPK: – AMP activated protein kinase;
ASD: – autism spectrum disorder;
ATG: – autophagy-related;
BPAN: – β-propeller protein-associated neurodegeneration;
CMA: – chaperone-mediated autophagy;
CNS: – central nervous system;
ER: – endoplasmic reticulum;
FXS: – Fragile X syndrome;
HSP: – hereditary spastic paraplegia;
ID: – intellectual disability;
IM: – isolation membrane;
KO: – knockout;
LAMP2: – lysosome-associated membrane protein 2;
LC: – light chain;
LDD: – Lhermitte-Duclos disease;
mTOR: – mechanistic target of rapamycin;
mTORC: – mTOR complex;
NSC: – neuronal stem cell;
PI3K: – phoshatidylinositol 3- kinase;
PI3P: – phosphatidylinositol 3-phosphate;
POMC: – proopiomelanocortin;
TSC: – tuberous sclerosis complex;
ULK1: – UNC51-like kinase 1.
